# A Survey of Viewpoint Selection Methods for Polygonal Models

**DOI:** 10.3390/e20050370

**Published:** 2018-05-16

**Authors:** Xavier Bonaventura, Miquel Feixas, Mateu Sbert, Lewis Chuang, Christian Wallraven

**Affiliations:** 1 Graphics & Imaging Laboratory, University of Girona, Girona 17003, Spain; 2 School of Computer Science and Technology, Tianjin University, Tianjin 300350, China; 3 Max Planck Institute for Biological Cybernetics, Tuebingen 72076, Germany; 4 Department of Brain Cognitive Engineering, Korea University, Seoul 02841, Korea

**Keywords:** visualization, viewpoint selection, entropy, mutual information

## Abstract

Viewpoint selection has been an emerging area in computer graphics for some years, and it is now getting maturity with applications in fields such as scene navigation, scientific visualization, object recognition, mesh simplification, and camera placement. In this survey, we review and compare twenty-two measures to select good views of a polygonal 3D model, classify them using an extension of the categories defined by Secord et al., and evaluate them against the Dutagaci et al. benchmark. Eleven of these measures have not been reviewed in previous surveys. Three out of the five short-listed best viewpoint measures are directly related to information. We also present in which fields the different viewpoint measures have been applied. Finally, we provide a publicly available framework where all the viewpoint selection measures are implemented and can be compared against each other.

## 1. Introduction

Why is viewpoint selection important? A large number of 3D models or objects are daily used across diverse fields such as computer game development, computer-aided design, and interior design. These models are often obtained by exploring large 3D model databases in as little time as possible. In this case, automated viewpoint selection plays an important role since such an application can show the model view that allows for ready recognition or understanding of the underlying 3D model. An ideal view should strive to capture the maximum information of the 3D model, such as its main characteristics, parts, functionalities, etc. The quality of this view could affect the number of models that the artist can explore in a certain period of time.

In the viewpoint selection study, the basic question is “what are good views of a 3D object or a scene?” In order to address this, a number of computational measures have been proposed to quantify the goodness or the quality of a view. Depending on our goals, the best viewpoint can be, for instance, the view that allows us to see the largest number of parts of the object, the view that shows the most salient regions of the object, or the view that maximally changes when the underlying object is jittered.

The human visual system is classically described [1] either in terms of its ability to recognize familiar three-dimensional objects as structural representations of their comprising part-components [2], or as multiple-view descriptions [3,4,5]. Biederman [2] proposed that familiar object recognition can be conceptualized as a computational process by which the projected retinal image of the three-dimensional object is segmented at regions of deep concavity to derive a reduced representation of its simple geometric components (e.g., blocks, cylinders, wedges, and cones) and their spatial relations. Nonetheless, many studies have since demonstrated that the visual system demonstrates preferential behavioral and neuronal responses to particular object views [5,6,7]. Indeed, recognition behavior continues to be highly selective for previously learned views even when highly unique object parts with little self-occlusion are made available for discrimination [6]. Naturally, this raises the question of which view(s) could represent a given object, so as to support robust visual recognition. Palmer et al. [8] found that participants tend to agree on the canonical view (or most representative image) of each familiar object that would facilitate its recognition.

They are often off-axis views, such as a top-down three-quarter view, which arguably reveals the largest amount of surface area. In contrast, Harman et al. [9] allowed participants to learn novel 3D objects by exploring them in virtual reality. They found that their participants spent time exploring “plan” views, namely views that were on-axis or orthogonal and parallel to the object’s structural axis. Perrett et al. [10,11] found a similar preference for “plan” views in tool-like as well as “novel” objects. The mixed evidence could be due to the fact that view-canonicity can be expressed by the multiple factors [12]: goodness for recognition (a good view for recognition shows the most salient and significant features, and it is stable with respect to small transformations, and it avoids a high number of occluded features), familiarity (recognition is influenced by the views that are encountered more frequently and during the initial learning), functionality (recognition is influenced by the views that are most relevant for how we interact with an object), and aesthetic criteria (preferred views can be influenced by geometric proportions).

In this survey, the computational measures that will be reviewed are those that were motivated for “goodness for recognition” instead of other aspects such as familiarity and aesthetics. The main contribution of this survey lies in collecting and testing in a common framework the most basic measures introduced to select the best views for polygonal models. We include most of the measures presented in previous surveys of viewpoint selection [13,14,15], but we do not consider semantic-based viewpoint selection measures, the absolute Gaussian and mean curvature [15], and the topological complexity [13]. In addition, we review eleven viewpoint selection measures that have not been included in the previous surveys.

This survey is organized as follows. In Section 2, we review pioneering work in view-selection and the basic measures that have been proposed for estimating the quality of views. In Section 3, the most relevant measures are defined and described. In Section 4, we test the presented measures using the Dutagaci et al. [14] benchmark. In Section 5, we present literature that applies the viewpoint quality measures to other fields of research. Finally, in Section 6, our conclusions and future work are presented.

## 2. Background

In this section, we present the basis of viewpoint selection, that is, landmark research and the most basic measures that gave rise to the other measures and methods that have also been used in the last decade (Section 3).

First, we review pioneer work on viewpoint selection. Attneave [16] analyzes informational aspects of visual perception and explains that information for object discrimination is concentrated along an object’s contour shape (i.e., 2D silhouette), especially where such information changes rapidly (i.e., peaks of curvature). Connolly [17] describes two algorithms that use partial octree models to determine the next best view to take. Kamada and Kawai [18] presented a measure to select a good view based on the angle between the view direction and the normal of the planes of the model. This method triest to avoid degenerative views, views where a plane is projected as a line and a line is projected as a point. Plemenos and Benayada [19] extended Kamada’s work to ensure that the user sees a great number of details. Plemenos’ measure takes into account the projected area and the number of polygons to evaluate the viewpoint goodness. Arbel and Ferrie [20] applied Shannon entropy to define entropy maps to guide an active observer along an optimal trajectory. Inspired by Kamada’s and Plemenos’ works, Vázquez et al. [21] also used the Shannon entropy to quantify the information provided by a view. This measure incorporates both the projected area and the number of faces.

Weinshall and Werman [22] define two measures: view likelihood and view stability. View likelihood measure is used to identify “characteristic” views based on the probability that a certain view of a given 3D object is observed. View stability is used to identify “generic” views based on how the image changes as the viewpoint is slightly modified. Stoev and Straßer [23] noticed that the projected area was not enough to visualize terrains and they presented a method that maximizes the maximum depth of the image in addition to the projected area. Given a sphere of viewpoints, Yamauchi et al. [24] computed the similarity between each two disjoint views using Zernike moments analysis and obtained a similarity weighted spherical graph. Here, a view was considered to be stable if all of the edges that were incident on its viewpoint in the spherical graph had high similarity weights.

Itti et al. [25] maintain that visual attention is saliency-dependent and use a saliency map to represent the conspicuity or saliency at every location in the visual field by a scalar quantity. Thus, a good view could be described as one that is likely to be attended to, given its high saliency content. Borji and Itti [26] presented a state-of-the-art in visual attention modeling that can compute saliency maps from any image or video input. From surface curvature, Lee et al. [27] introduced a perception-inspired measure of regional importance, called mesh saliency, that has been used in mesh simplification and viewpoint selection. Gal and Cohen-Or [28] introduced a method for partial matching of surfaces by using the abstraction of salient geometric features and a method to construct them.

Some measures that consider semantic information of the model have been also used in viewpoint selection. High-level and semantic measures take into account features such as the topology of the model, the position of the eyes, or the part used to grasp the object. Becker et al. [29] analyze how object-intrinsic oddities can be detected by previous semantic knowledge of the object, and that draw the attention of the viewer by its oddity. Koulieris et al. [30] define a high-level saliency model for objects within a scene, based on singletoness and semantic coherence with environment objects, that allow to identify objects to be rendered in a higher detail. To include this saliency model in the view point selection process, a priori semantic information about the objects constituting the scene is needed. Secord et al. [15], based on the work of Blanz et al. [12] and Gooch et al. [31], propose a measure that captures views from slightly above the horizon. Secord et al. [15] also introduced a measure that tends to avoid views from directly below for objects that have an obvious orientation. The automatic method of Fu et al. [32] can be used to determine both the base and the orientation of the object. When the model is a creature with eyes or a face, people prefer views where the eyes are visible [33]. Secord et al. [15] have further proposed an attribute that sums all the visible pixels corresponding to the eyes’ surface. Finally, it is worth mentioning that Podolak et al. [34] have introduced a method to choose good viewpoints automatically by minimizing the symmetry of the object seen from the viewpoint.

Polonsky et al. [13] and Secord et al. [15] have described and analyzed a number of measures that were introduced to quantify the goodness of a view of an object. After analyzing different view descriptors, Polonsky et al. [13] concluded that no single descriptor does a perfect job and have suggested that a combination of descriptors would amplify their respective advantage over each other. In this regard, Secord et al. [15] have presented a perceptual model of viewpoint selection based on the combination of different attributes such as surface visibility, silhouette length, projected area, and maximum depth. If the region corresponding to the eyes’ surface is marked, Secord et al. [15] have proposed changing the maximum depth according to eye preference.

Dugataci et al. [14] have presented a benchmark to validate best view selection methods by analyzing the accuracy of these methods in comparison with the preferred views selected by 26 human subjects. In this benchmark, the human subjects were asked to select the most informative view of 68 3D models through a web page. Dugataci et al. [14] also compute for every model the inconsistency of the choices of the human subjects. They provide a way to quantify the error of a best view selection algorithm compared to the data collected. An error between 0 and 1 and the average for all the models can be computed using the benchmark. To compute the error, they take into account the symmetry of the models. Most of the models used in this benchmark are common objects highly familiar to humans. The benchmark was tested with seven different methods computed in a sphere of 258 viewpoints. The methods tested were view area, ratio of visible area, surface area entropy, silhouette length, silhouette entropy, curvature entropy, and mesh saliency.

## 3. Viewpoint Selection Measures

In this section, we gather twenty-two viewpoint selection measures that are classified according to several attributes captured from a particular viewpoint: area, silhouette, depth, stability, and surface curvature. These categories, except stability, are presented in Secord et al. [15]. For each measure, we provide its definition and the reference of the paper where the measure was introduced. All the measures presented in this section will be tested in Section 4 and are available in a public common framework.

### 3.1. Notation

For comparison purposes, we propose a unified notation for the analyzed measures adopted from Feixas et al. [35], where an information channel was defined between a set of viewpoints V and a set of polygons Z. The projected area of polygon *z* from viewpoint *v* is denoted by $a_{z}\left(v\right)$ and the projected area of the model from viewpoint *v* is given by $a_{t}\left(v\right)$. Viewpoint quality *v* is expressed by VQ(v).

In Feixas et al. [35], a viewpoint selection framework was proposed from an information channel V→Z between the random variables *V* (input) and *Z* (output), which represent, respectively, a set of viewpoints V and the set of polygons Z of an object. This channel is defined by a conditional probability matrix obtained from the projected areas of polygons at each viewpoint and can be interpreted as a visibility channel where the conditional probabilities represent the probability of seeing a determined polygon from a given viewpoint. The three basic elements of the visibility channel are:

- Conditional probability matrix p(Z|V), where each element $p\left(z|v\right)=\frac{a_{z}\left(v\right)}{a_{t}\left(v\right)}$ is defined by the normalized projected area of polygon *z* over the sphere of directions centered at viewpoint *v*.
  Conditional probabilities fulfill $\sum_{z\in Z}p\left(z|v\right)=1$.
- Input distribution p(V), where each element $p\left(v\right)=\frac{a_{t}\left(v\right)}{\sum_{v\in V}a_{t}\left(v\right)}$, which represents the probability of selecting each viewpoint, is obtained from the normalization of the object projected area at each viewpoint. The input distribution is interpreted as the importance assigned to each viewpoint *v*.
- Output distribution p(Z), given by $p\left(z\right)=\sum_{v\in V}p\left(v\right)p\left(z|v\right)$, which represents the average projected area of polygon *z*.

Table 1 and Table 2 show, respectively, the notation used in the measure definitions and the list of measures studied in this paper. Observe that Table 2 also contains additional information for each measure. Columns 3, 4, and 5 show the corresponding names used in surveys by Polonsky et al. [13], Dugataci et al. [14], and Secord et al. [15], respectively. Column 6 indicates whether the best viewpoint corresponds to the highest (H) or the lowest (L) measure value. Column 7 shows whether the measure is sensitive (Y) to how the polygonal model is discretized or not (N). In addition, column 8 gives the main reference of the measure presented.

### 3.2. Area Attributes

The measures based on these attributes are computed using as main feature the area of polygons seen from a particular viewpoint.

**Number of visible triangles.** Plemenos and Benayada [19] used the number of visible triangles seen from a viewpoint as a viewpoint quality measure. The higher the number of visible triangles, the better the quality of a viewpoint. This measure is based on the fact that the most significant regions contain more details and, thus, more triangles. This measure is expressed as

(1) $$VQ_{1}\left(v\right)=\sum_{z\in Z}vis_{z}\left(v\right),$$

where $vis_{z}\left(v\right)$ is 1 if the polygon *z* is visible from viewpoint *v* and 0 otherwise. Different criteria can be used to consider whether a polygon is visible. In our implementation, a polygon is considered visible if at least one pixel of polygon *z* is visible from viewpoint *v* ($a_{z}\left(v\right)>0$). Obviously, the number of visible triangles is sensitive to the discretization of the model.

**Projected area.** Plemenos and Benayada [19] also studied the projected area of the model from a viewpoint as a measure of viewpoint goodness since the number of visible triangles was found not to be enough in some cases. For example, if we consider a pencil, it is normal to have a high number of polygons around the pencil point. If we use the number of visible triangles to select the best viewpoint, we would only see a small part of the object. The projected area expressed as

(2) $$VQ_{2}\left(v\right)=a_{t}\left(v\right)$$

can be considered as a viewpoint quality measure. Thus, the higher the projected area, the better the viewpoint quality. This measure is insensitive to the discretization of the model.

**Plemenos and Benayada.** Plemenos and Benayada [19] combined the number of visible triangles and the projected area to create a measure for viewpoint quality. A viewpoint is considered good if the percentage of the number of visible polygons plus the percentage of projected area with respect to the size of the screen is high. This measure can be expressed as

(3) $$VQ_{3}\left(v\right)=\frac{\sum_{z\in Z}\left\lceil \frac{a_{z}\left(v\right)}{a_{z}\left(v\right)+1}\right\rceil }{N}+\frac{\sum_{z\in Z}a_{z}\left(v\right)}{R},$$

where *R* is the total number of pixels of the image and *N* the total number of polygons (i.e., N=|Z|). For more details, see also Barral et al. [36]. Note that the first term is the ratio of visible polygons, where $\left\lceil \frac{a_{z}\left(v\right)}{a_{z}\left(v\right)+1}\right\rceil$ is equivalent to $vis_{z}\left(v\right)$, and the second term is the ratio of the projected area with respect to the resolution of the screen. Thus, $VQ_{3}\left(v\right)$ can be rewritten as

(4) $$VQ_{3}\left(v\right)=\frac{VQ_{1}\left(v\right)}{N}+\frac{VQ_{2}\left(v\right)}{R}.$$

This measure is sensitive to polygonal discretization because $VQ_{1}\left(v\right)$ is, as we have seen above.

**Visibility ratio.** Plemenos and Benayada [19] also introduced the ratio between the visible surface area of the model from viewpoint *v* and the total surface area as a viewpoint quality measure. The visibility ratio is expressed by

(5) $$VQ_{4}\left(v\right)=\frac{\sum_{z\in Z}vis_{z}\left(v\right)A_{z}}{A_{t}},$$

where $A_{z}$ is the area of polygon *z*, and $A_{t}$ is the total area of the model. Observe that $A_{z}$ does not depend on the viewpoint because denotes the real area of polygon *z*. The best viewpoint corresponds to the minimum value of the measure. This measure is insensitive to the discretization of the model.

**Viewpoint entropy.** Vázquez et al. [21,37] presented a measure for viewpoint selection based on Shannon entropy [38,39]. This measure takes into account the projected area and the number of viewpoints and can be understood as the amount of information captured by a specific viewpoint. The viewpoint entropy is defined by

(6) $$VQ_{5}\left(v\right)=H\left(v\right)=-\sum_{z\in Z}\frac{a_{z}\left(v\right)}{a_{t}\left(v\right)}\log\frac{a_{z}\left(v\right)}{a_{t}\left(v\right)}.$$

Using the notation of the visibility channel introduced in Section 3.1, the viewpoint entropy is rewritten as

(7) $$VQ_{5}\left(v\right)=H\left(Z|v\right)=-\sum_{z\in Z}p\left(z|v\right)\log p\left(z|v\right),$$

where H(Z|v) represents the conditional entropy of *Z* given a viewpoint *v*. The best viewpoint corresponds to the one with maximum entropy, which is obtained when a certain viewpoint can see all the faces with the same relative projected area. Viewpoint entropy is sensitive to polygonal discretization as in general the entropy increases with the number of polygons.

Polonsky et al. [13] propose the application of viewpoint entropy using the probability of semantically important segments of the model.

**Information $I_{2}$.** Deweese and Meister [40] used a decomposition of mutual information in the field of neuroscience to quantify the information associated with stimuli and responses. Bonaventura et al. [41] applied this measure to the field of best viewpoint selection to express the informativeness of a viewpoint. The viewpoint information $I_{2}$ is defined by

(8) $$\begin{array}{ccc} VQ_{6}\left(v\right)=I_{2}\left(v;Z\right) & = & H(Z)-H(Z|v)\\ & = & H\left(Z\right)-VQ_{5}\left(v\right)\\ & = & -\sum_{z\in Z}p\left(z\right)\log p\left(z\right)+\sum_{z\in Z}p\left(z|v\right)\log p\left(z|v\right), \end{array}$$

where H(Z) stands for the entropy of model triangles. Note that $I_{2}$ is closely related to viewpoint entropy, defined as H(Z|v) [21,35], since $I_{2}\left(v;Z\right)=H\left(Z\right)-H\left(Z|v\right)$. As H(Z) is constant for a given mesh resolution, $I_{2}\left(v;Z\right)$ and viewpoint entropy have the same behavior in viewpoint selection because the highest value of $I_{2}\left(v;Z\right)$ corresponds to the lowest value of viewpoint entropy, and vice versa. An important drawback of viewpoint entropy is that it goes to infinity for finer and finer resolutions of the mesh [35], while $I_{2}$ presents a more stable behavior due to the normalizing effect of H(Z) in Equation (8). The best viewpoint is given by the one that has minimum $I_{2}$. Similarly to viewpoint entropy, this measure is also sensitive to polygonal discretization.

**Viewpoint Kullback–Leibler distance (VKL).** Sbert et al. [42] presented a viewpoint quality measure given by the Kullback–Leibler distance between the normalized distribution of the projected areas of polygons from viewpoint *v* and the normalized distribution of the real areas of polygons. The viewpoint Kullback–Leibler distance is given by

(9) $$VQ_{7}\left(v\right)=\sum_{z\in Z}\frac{a_{z}\left(v\right)}{a_{t}\left(v\right)}\log\frac{\frac{a_{z}\left(v\right)}{a_{t}\left(v\right)}}{\frac{A_{z}}{A_{t}}}.$$

Observe that the minimum value, which corresponds to the best viewpoint, is obtained when the normalized distribution of projected areas is equal to the normalized distribution of real areas. Viewpoint Kullback–Leibler distance is near insensitive to polygonal discretization.

**Viewpoint mutual information (or $I_{1}$).** Feixas et al. [35] presented a measure, called viewpoint mutual information (VMI), that captures the degree of correlation between a viewpoint and the set of polygons. Bonaventura et al. [41] renamed this measure as $I_{1}$ because this is one of the decomposition forms of mutual information used to deal with stimuli and responses [40]. The viewpoint mutual information is defined by

(10) $$VQ_{8}\left(v\right)=VMI\left(v\right)=I_{1}\left(v;Z\right)=\sum_{z\in Z}p\left(z|v\right)\log\frac{p(z|v)}{p(z)}.$$

High values of the measure mean a high correlation between viewpoint *v* and the object, indicating a highly coupled view (for instance, between the viewpoint and a small number of polygons with low average visibility). On the other hand, the lowest values correspond to the most representative or relevant views (i.e., best viewpoints), showing the maximum possible number of polygons in a balanced way. VMI is insensitive to the discretization of the model. For more information, see [43].

**Information $I_{3}$.** Butts [44] introduced a new decomposition form of mutual information, called $I_{3}$, to quantify the specific information associated with a stimulus. Bonaventura et al. [41] proposed $I_{3}$ as a viewpoint quality measure. The measure $I_{3}$ is defined by

(11) $$\begin{array}{c} VQ_{9}\left(v\right)=I_{3}\left(v;Z\right)=\sum_{z\in Z}p\left(z|v\right)I_{2}\left(V;z\right), \end{array}$$

where $I_{2}\left(V;z\right)$ is the specific information of polygon *z* given by

(12) $$\begin{array}{ccc} I_{2}\left(V;z\right) & = & H(V)-H(V|z)\\ & = & -\sum_{v\in V}p\left(v\right)\log p\left(v\right)+\sum_{v\in V}p\left(v|z\right)\log p\left(v|z\right), \end{array}$$

where $p\left(v|z\right)=\frac{p(v)p(z|v)}{p(z)}$ (Bayes theorem). Note that H(V) and H(V|z) represent the entropy of the set of viewpoints and the conditional entropy of the set of viewpoints given polygon *z*, respectively. A high value of $I_{3}\left(v;Z\right)$ means that the polygons seen by *v* are very informative in the sense of $I_{2}\left(V;z\right)$. The most informative viewpoints are considered as the best views and correspond to the viewpoints that see the highest number of maximally informative polygons. The measure $I_{3}$ is sensitive to polygonal discretization.

### 3.3. Silhouette Attributes

The measures based on these attributes are computed using the silhouette of the object seen from a particular viewpoint. All these measures are insensitive to the discretization of the model because the polygons are not directly used.

**Silhouette length.** Polonsky et al. [13] presented the silhouette length of the projected model from a viewpoint *v* as a measure of viewpoint goodness. The silhouette length is expressed as

(13) $$VQ_{10}\left(v\right)=slength\left(v\right),$$

where slength(v) stands for the silhouette length from *v*. In our implementation, the silhouette length of the model is computed from the viewpoint *v* by counting the number of pixels that belong to the silhouette. If there are multiple contours, the pixels of all the contours are added. The goodness of a viewpoint is associated with the maximum silhouette length.

**Silhouette entropy.** Polonsky et al. [13] introduced the entropy of the silhouette curvature distribution, proposed by Page et al. [45], as a measure of viewpoint goodness. In our implementation, the silhouette curvature histogram is computed from the turning angles between consecutive pixels belonging to the silhouette. The range of the curvature is between −π/2 and π/2 with a step of π/4 due to the angles obtained between neighbor pixels. The silhouette entropy is defined by

(14) $$VQ_{11}\left(v\right)=-\sum_{\alpha =-\pi /2}^{\pi /2}h\left(\alpha \right)\log h\left(\alpha \right),$$

where {h(α)} represents the normalized silhouette curvature histogram and α is the turning angle bin. The best viewpoint is the one with the highest silhouette entropy.

**Silhouette curvature.** Vieira et al. [46] introduced the complexity of the silhouette defined as the total integral of its curvature. In our implementation, the silhouette curvature is computed as

(15) $$VQ_{12}\left(v\right)=\frac{\sum_{c\in C}\frac{\left|c\right|}{\frac{\pi }{2}}}{N_{c}},$$

where *c* is the turning angle between two consecutive pixels, C is the set of turning angles, and $N_{c}$ is the number of turning angles, equal to the number of pixels of the silhouette. The best viewpoint is given by the one with the maximum value.

**Silhouette curvature extrema.** As a variation of the above silhouette curvature measure, Secord et al. [15] introduced the silhouette curvature extrema to emphasize high curvatures on the silhouette. The silhouette curvature extrema is computed as

(16) $$VQ_{13}\left(v\right)=\frac{\sum_{c\in C}{\left(\frac{c}{\frac{\pi }{2}}\right)}^{2}}{N_{c}}.$$

Similarly to silhouette curvature, the higher the value, the better the viewpoint.

### 3.4. Depth Attributes

The measures based on these attributes are computed using the depth of the model seen from a particular viewpoint.

**Stoev and Straßer.** Stoev and Straßer [23] noticed that the projected area was not enough to visualize terrains because usually the view with most projected area is the one from above. They presented a method for camera placement that maximizes the maximum depth of the image in addition to the projected area. This measure is defined by

(17) $$VQ_{14}\left(v\right)=\alpha p\left(v\right)+\beta d\left(v\right)+\gamma \left(1-|d\left(v\right)-p\left(v\right)|\right),$$

where p(v) is the normalized projection area from viewpoint *v* and d(v) is the normalized maximum depth of the scene from viewpoint *v*. For general purposes, the authors proposed the use of the following values: $\alpha =\beta =\gamma =\frac{1}{3}$. The Stoev and Straßer measure used in our implementation is given by

(18) $$VQ_{14}\left(v\right)=\frac{1}{3}p\left(v\right)+\frac{1}{3}d\left(v\right)+\frac{1}{3}\left(1-|d\left(v\right)-p\left(v\right)|\right).$$

For terrain scenarios, Stoev and Straßer [23] considered $\alpha =\beta =\frac{1}{4}$ and $\gamma =\frac{1}{2}$. The best viewpoint is the one with the maximum value, maximizing the projected area and the maximum depth and minimizing the difference between the projected area and the maximum depth. This measure is insensitive to polygonal discretization because the projected area and the maximum depth are insensitive too.

**Maximum depth.** Secord et al. [15] considered only the maximum depth, used in Stoev and Straßer [23], as a descriptor of viewpoint quality. This measure is thus defined as

(19) $$VQ_{15}\left(v\right)=depth\left(v\right),$$

where depth(v) is the maximum depth. As we have seen above, the maximum depth is insensitive to polygonal discretization and the best viewpoint is considered as the one with the maximum value.

**Depth distribution.** Instead of using only the maximum depth from a viewpoint, Secord et al. [15] proposed a measure that maximizes the visible range of depths. The depth distribution measure defined by

(20) $$VQ_{16}\left(v\right)=1-\sum_{d\in D}h{\left(d\right)}^{2}$$

tries to capture the maximum diversity of depths, where *d* represents a depth bin, D is the set of depth bins, and {h(d)} the normalized histogram of depths. The best viewpoint corresponds to the maximum value of the measure. This measure is insensitive to the discretization of the model.

### 3.5. Stability Attributes

The measures based on these attributes compute the stability of a viewpoint by comparing the viewpoint with its neighbors.

**Instability.** Feixas et al. [35] defined viewpoint instability from the notion of dissimilarity between two viewpoints, which is given by the Jensen–Shannon divergence [47] between their respective projected area distributions. The use of Jensen–Shannon as a measure of view similarity was proposed by Bordoloi and Shen [48] in the volume rendering field. The viewpoint instability of *v* is defined by

(21) $$VQ_{17}\left(v\right)=\frac{1}{N_{v}}\sum_{j=1}^{N_{v}}D\left(v,v_{j}\right),$$

where $v_{j}$ is a neighbor of *v*, $N_{v}$ is the number of neighbors of *v*, and

$$D\left(v,v_{j}\right)=JS\left(\frac{p(v)}{p\left(v\right)+p\left(v_{j}\right)},\frac{p(v_{j})}{p\left(v\right)+p\left(v_{j}\right)};p\left(Z|v\right),p\left(Z|v_{j}\right)\right)$$

is the Jensen–Shannon divergence between the distributions p(Z|v) and $p(Z|v_{j})$ captured by *v* and $v_{j}$ with weights $\frac{p(v)}{p\left(v\right)+p\left(v_{j}\right)}$ and $\frac{p(v_{j})}{p\left(v\right)+p\left(v_{j}\right)}$, respectively. The best viewpoint is the one with the lowest instability. The instability measure is sensitive to the discretization of the model.

**Depth-based visual stability.** Vázquez [49] introduced a method to compute the view stability from the depth images of all viewpoints. The degree of similarity between two viewpoints is given by the normalized compression distance (NCD) between two depth images:

(22) $$similarity\left(v_{i},v_{j}\right)=NCD\left(v_{i},v_{j}\right)=\frac{L\left(v_{i}v_{j}\right)-min\left\{L\left(v_{i}\right),L\left(v_{j}\right)\right\}}{max\left\{L\left(v_{i}\right),L\left(v_{j}\right)\right\}},$$

where $L(v_{i})$ and $L(v_{j})$ are, respectively, the sizes of the compression of the depth images corresponding to viewpoints $v_{i}$ and $v_{j}$, and $L(v_{i}v_{j})$ is the size of the compression of the concatenation of the depth images corresponding to $v_{i}$ and $v_{j}$.

Two views are considered similar if their distance is less than a given threshold. Hence, the most stable view is given by the one that has the largest number of similar views. The depth-based visual stability is given by

(23) $$VQ_{18}\left(v\right)=\#similar\;views\;to\;v.$$

This measure is robust to the discretization of the model because an image-based method is used. However, it is highly sensitive to the threshold value. The best view corresponds to the most stable one.

### 3.6. Surface Curvature Attributes

The measures based on these attributes are computed using the surface curvature of the shape. Note that, in the last two measures (Equations (28) and (29)), area attributes are also taken into account.

**Curvature entropy.** Polonsky et al. [13] propose a measure that evaluates the entropy of the curvature distribution over the visible portion of surface from a given viewpoint. This measure is inspired by the entropy of the Gaussian curvature distribution defined by Page et al. [45]. The curvature of vertex *i* is defined by

(24) $$K_{i}=2\pi -\sum_{j}\phi_{j},$$

where the angle $\phi_{j}$ is the wedge subtended by the edges of a triangle whose corner is at the vertex *i*. The curvature entropy of a viewpoint *v* is defined by

(25) $$VQ_{19}\left(v\right)=-\sum_{b\in B}h\left(b\right)\log h\left(b\right),$$

where *b* represents a curvature bin, B is the set of curvature bins, and {h(b)} the normalized histogram of visible curvatures from viewpoint *v*. The higher the value, the better the viewpoint. Curvature entropy is sensitive to the discretization of the model.

**Visible saliency.** Lee et al. [27] presented a measure to select the best viewpoint based on the amount of saliency seen from a viewpoint. The saliency used is presented by Lee et al. [27] and it is computed for every vertex using the curvature presented by Taubin [50]. The visible saliency measure is the sum of all the saliences of the vertices seen from viewpoint *v* and is defined by

(26) $$VQ_{20}\left(v\right)=\sum_{x\in X}S\left(x\right),$$

where X is the set of visible vertices and S(x) the saliency of vertex *x*. The saliency of vertex *x* is defined by

(27) S(x)=|G(C(x),σ)−G(C(x),2σ)|,

where G(C(v),σ) is the Gaussian-weighted average of the mean curvature. The higher the value, the better the viewpoint. Visual saliency is sensitive to polygonal discretization since the summation is done for the visible vertices. Similarly to Lee et al. [27], Sokolov and Plemenos [51] present a viewpoint quality measure given the sum of curvatures captured by a viewpoint where the curvature is computed as in Equation (24).

**Projected saliency.** Inspired by the visual saliency [27], Feixas et al. [35] presented a method to select the best view using the saliency of the polygons. This saliency is computed for every polygon using an information channel between polygons and viewpoints. The projected saliency is defined by

(28) $$VQ_{21}\left(v\right)=\sum_{z\in Z}S\left(z\right)p\left(v|z\right),$$

where S(z) is saliency of polygon *z* computed as

$$S\left(z\right)=\frac{1}{N_{z}}\sum_{j=1}^{N_{z}}D\left(z,z_{j}\right),$$

where polygon $z_{j}$ is a neighbor of polygon *z*, $N_{z}$ is the number of neighbors of *z*, and

$$D\left(z,z_{j}\right)=JS\left(\frac{p(z)}{p\left(z\right)+p\left(z_{j}\right)},\frac{p(z_{j})}{p\left(z\right)+p\left(z_{j}\right)};p\left(V|z\right),p\left(V|z_{j}\right)\right)$$

is the Jensen–Shannon divergence between the distributions p(V|z) and $p(V|z_{j})$ with weights $\frac{p(z)}{p\left(z\right)+p\left(z_{j}\right)}$ and $\frac{p(z_{j})}{p\left(z\right)+p\left(z_{j}\right)}$, respectively. The higher the value the better the viewpoint. The projected saliency is sensitive to the discretization of the model. Similarly, other polygonal information measures have been projected to the viewpoints to select a good view [52].

**Saliency-based EVMI.** Feixas et al. [35] presented an extended version of viewpoint mutual information (EVMI) where the target distribution is weighted by an importance factor. The importance-based EVMI is defined by

(29) $$VQ_{22}\left(v\right)=\sum_{z\in Z}p\left(z|v\right)\log\frac{p(z|v)}{p^{\prime }\left(z\right)},$$

where $p^{\prime }\left(z\right)$ is given by

(30) $$p^{\prime }\left(z\right)=\frac{p(z)i(z)}{\sum_{z\in Z}p\left(z\right)i\left(z\right)},$$

where i(z) is the importance of polygon *z*. The saliency-based EVMI is obtained when i(z)=S(z) [35]. Similarly to VMI, the best viewpoint corresponds to the minimum value. Saliency-based EVMI is sensitive to polygonal discretization because the saliency of a polygon is sensitive too. Serin et al. [53] presented a similar measure where i(z) is given by the surface curvature and p(z) (i.e., average projected area) is substituted by the total area of the polygon.

## 4. Results and Discussion

In this section, we test and compare the measures presented in Section 3.2, Section 3.3, Section 3.4, Section 3.5 and Section 3.6. These measures are computed for every model without considering any semantic information, such as the object’s preferred orientation. First, we describe the details of the implementation used to compute the viewpoint selection measures. Second, we illustrate for all the measures the best view of three different 3D models. Third, the Dutagaci et al. [14] benchmark is used to analyze the accuracy of these measures in comparison with the best views selected by 26 human subjects. The presented measures, except the visual saliency measure, have been implemented in a common framework. For the visual saliency measure ($VQ_{20}$), we have used Dutagaci’s implementation [14]. This is the only measure not included in the framework.

To compute the projected area of a polygon (usually a triangle), we use a projection resolution of 640 × 640 pixels. No back-face culling optimization is applied and the polygons are rendered from both sides. All of the models are centered inside a sphere of 642 viewpoints built from the recursive discretization of an icosahedron, and the camera is looking at the center of this sphere. The radius of the viewpoint sphere is six times the radius of the smallest bounding sphere of the model, the perspective distortion being acceptable. The view-frustum of the camera ($19.2^{{}^{\circ}}$) is adjusted to ensure that only the model and the minimum background is seen. For the results of the depth-based visual stability measure ($VQ_{18}$), we use a projection resolution of 128 × 128 pixels to reduce the computation time. In this case, the threshold used to decide if two viewpoints are similar is 0.87. Our framework, including the source code, is available at [54]. In this framework, the user can add and test new measures.

**Figure 1:**
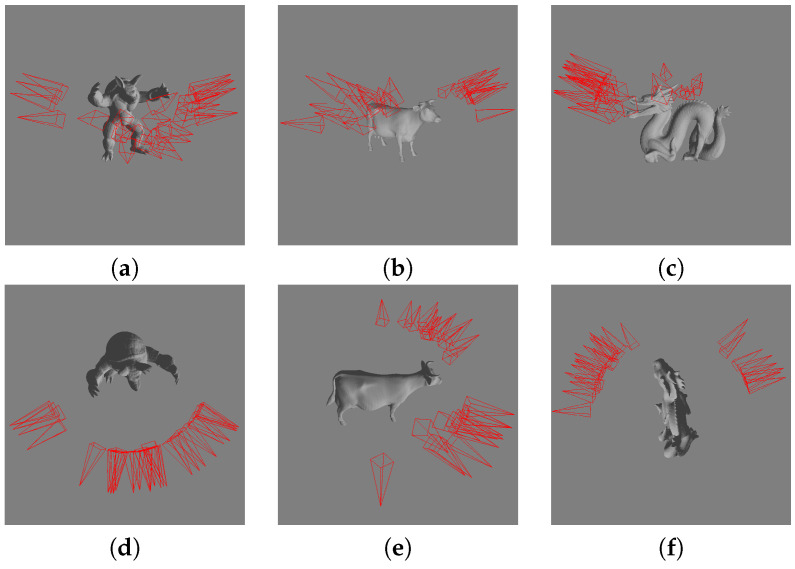
Set of best views for the armadillo, (**a**,**d**), the cow, (**b**,**e**), and the dragon, (**c**,**f**), selected by the 26 human subjects in the Dutagaci et al. [14] benchmark.

**Figure 2:**
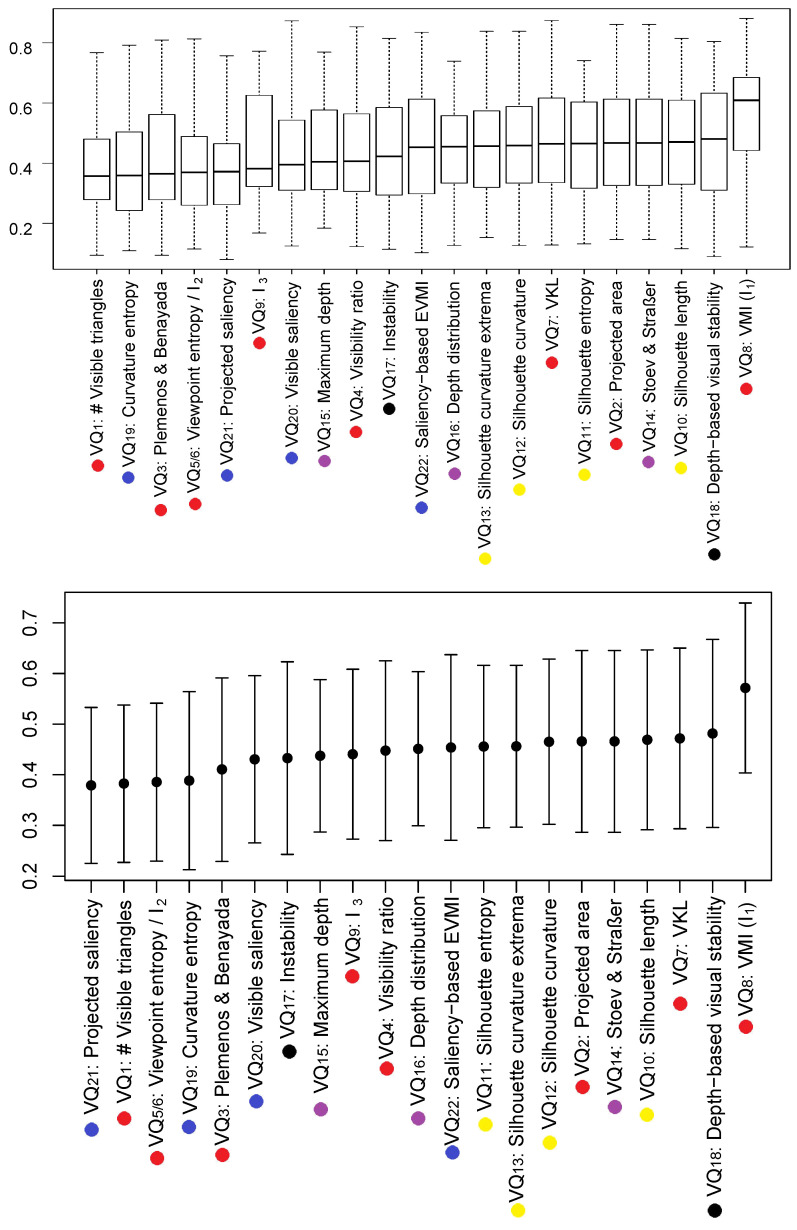
Box plot ordered by median (Top) and mean +/- standard deviation (Bottom) of the error of each method running the Dutagaci et al. [14] benchmark that checks 68 different models. The attribute category is marked with a color dot: area (red), silhouette (yellow), depth (purple), stability (black), and surface curvature (blue).

To show the goodness of the viewpoint quality measures, three 3D models of the Dutagaci benchmark are used: the Standford Armadillo (17,296 triangles), a cow (23,216 triangles), and the Standford dragon (26,142 triangles). Figure 1 shows the best views selected by 26 human subjects in the Dutagaci et al. [14] benchmark. Note that viewpoint entropy and information $I_{2}$ are grouped in Figure 2 and in the following reported results since they have the same performance (see Equation (8) in Section 3.2).

**Figure 3:**
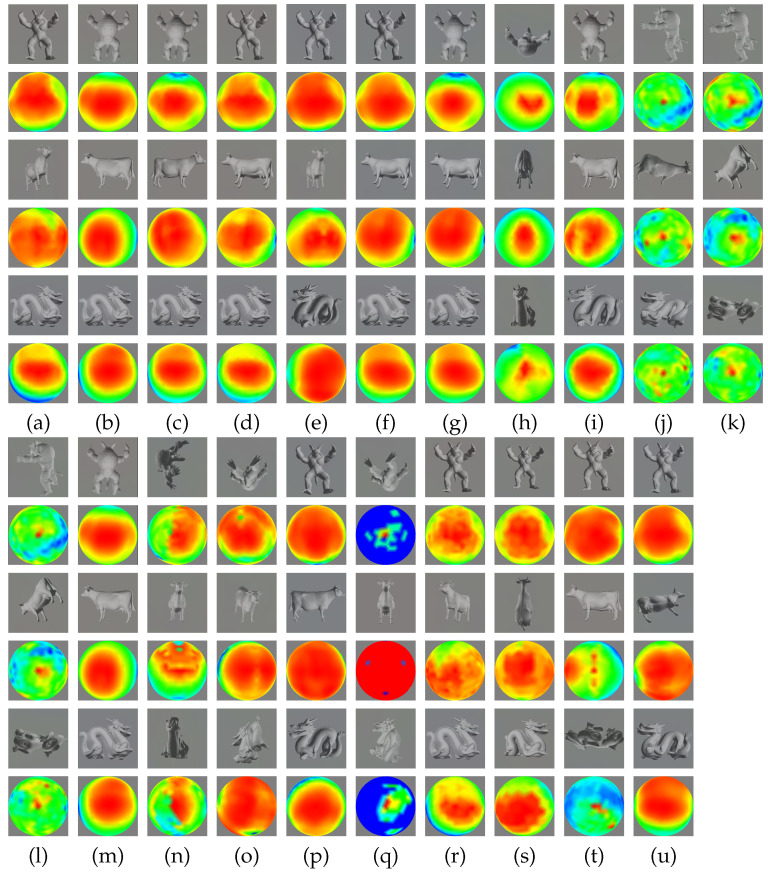
The best view and the corresponding sphere of viewpoints of three models using different methods: (**a**) $VQ_{1}$, # visible triangles; (**b**) $VQ_{2}$, projected area; (**c**) $VQ_{3}$, Plemenos and Benayada; (**d**) $VQ_{4}$, visibility ratio; (**e**) $VQ_{5}$, viewpoint entropy / $VQ_{6}$, $I_{2}$; (**f**) $VQ_{7}$, viewpoint Kullback–Leibler; (**g**) $VQ_{8}$, viewpoint mutual information ($I_{1}$); (**h**) $VQ_{9}$, $I_{3}$; (**i**) $VQ_{10}$, silhouette length; (**j**) $VQ_{11}$, silhouette entropy; (**k**) $VQ_{12}$, silhouette curvature; (**l**) $VQ_{13}$, silhouette curvature extrema; (**m**) $VQ_{14}$, Stoev and Straßer; (**n**) $VQ_{15}$, maximum depth; (**o**) $VQ_{16}$, depth distribution; (**p**) $VQ_{17}$, instability; (**q**) $VQ_{18}$, depth-based visual stability; (**r**) $VQ_{19}$, curvature entropy; (**s**) $VQ_{20}$, visual saliency; (**t**) $VQ_{21}$, projected saliency; and (**u**) $VQ_{22}$, saliency-based EVMI.

Figure 3 (from column (a) to column (u)) shows the best view and the corresponding viewpoint sphere obtained with the following viewpoint quality measures: (a) number of visible triangles, (b) projected area, (c) Plemenos and Benayada, (d) visibility ratio, (e) viewpoint entropy/$I_{2}$, (f) viewpoint Kullback–Leibler distance, (g) viewpoint mutual information (or $I_{1}$), (h) $I_{3}$, (i) silhouette length, (j) silhouette entropy, (k) silhouette curvature, (l) silhouette curvature extrema, (m) Stoev and Straßer, (n) maximum depth, (o) depth distribution, (p) instability, (q) depth-based visual stability, (r) curvature entropy, (s) visual saliency, (t) projected saliency, and (u) saliency-based EVMI. Rows (i), (iii), and (v) show, respectively, the best views of the armadillo, the cow, and the dragon, and rows (ii), (iv), and (vi) show the corresponding viewpoint sphere from the selected viewpoint. The sphere of viewpoints is represented by a color map, where red and blue colors correspond, respectively, to the best and worst viewpoints in terms of the corresponding viewpoint quality measures. From the different distributions, we can see the preferred and unfavored regions, the transition between them, and also the stability of the measure with respect to small viewpoint variations.

We evaluate the set of measures presented in Section 3 with Dutagaci’s benchmark [14]. This benchmark uses the most informative view of 68 models chosen by 26 human subjects. An error between 0 and 1 and the average for all the models can be computed using the benchmark. In Figure 2, we show the box plot ordered by median (top) and the mean +/- the standard deviation ordered by mean (bottom) of the error of the models for each method. We also mark the category of each measure with a color: area attribute (red), silhouette attribute (yellow), depth attribute (purple), stability attribute (black), and surface curvature attribute (blue). Observe that, if we rank the measures in terms of mean and median, the sets of the five best ones are the same: projected saliency [35], the number of visible triangles [19], viewpoint entropy and $I_{2}$ [21,41], curvature entropy [13], and Plemenos and Benayada [19]. Observe also that the five best measures belong to two categories: area attributes and surface curvature attributes. In contrast, the measures from the silhouette attributes category perform poorly. One reason for this could be returning to the idea that we represent objects in terms of volumetric primitives [55,56]. In this regard, area attributes and curvature could allow for the reconstruction of (or access to) higher-order representations than 2D image based properties. One could argue that it is not sufficient for the outline of an object to access mental representations of objects, but, rather, the outlines or properties that allow for the identification of element parts.

## 5. Applications

We present here some applications of the viewpoint quality measures of Section 3 to other fields of research. In Table 3, for each reference, we specify the measure(s) used or the measures the reference is inspired by, and the field of application. The fields of application considered in Table 3 are scene exploration and camera placement (SE/CP), image-based modeling and rendering (IBMR), scientific visualization (SV), shape retrieval (SR), and mesh simplification (MS). We only review the papers related to the measures presented in Section 3. Note also that some of the measures in Table 3 might not fully match the ones introduced in Section 3, but they are as closely related as to be considered under the same token.

Barral et al. [36,57] apply viewpoint quality measures to compute an efficient exploratory path for the visual understanding of a scene. Vázquez and Sbert [58] present a method for the automatic exploration of indoor scenes. They take into account the increase of information in terms of viewpoint entropy to decide the next position and orientation of the camera. Andújar et al. [59] present an algorithm for the automatic exploration of a scene. First, a cell-and-portal detection method identifies the over-all structure of the scene; second, an entropy-based measurement algorithm is used to identify the cells that are worth visiting, and third, a path is built that traverses all the relevant cells. Feixas et al. [35] present two object exploration algorithms based on viewpoint mutual information. In the first algorithm (guided tour), the path visits a set of N preselected best views, which ensures a good exploration of the object. In the second algorithm (exploratory tour), the successive viewpoints are selected using the maximum novelty criterion with respect to the parts seen of the object. Ozaki et al. [60] use viewpoint entropy to automatically generate a smooth movement of a camera to follow a subject. Serin et al. [61] present a viewpoint entropy-based approach to navigate over a 3D terrain. Best viewpoints for extracted subregions are calculated with a greedy N-best view selection algorithm.

Massios and Fisher [62] use the next best view for the reconstruction of a 3D object using a laser range scanner with the minimum number of viewpoints. Fleishman et al. [63] use the projected area to compute a minimum set of viewpoint inside a walking zone for image-based modeling. Vázquez et al. [37] use viewpoint entropy to minimize the number of images used for image-based rendering.

Bordoloi and Shen [48] compute the instability and the viewpoint entropy in volume rendering to select the best and the N best views of volumetric data. They also apply them to time-varying data. Takahashi et al. [64] apply viewpoint entropy to volume visualization by decomposing an entire volume into a set of feature components. Ji and Shen [65] implement a time-varying view for time-varying volumes in order to maximize the amount of information seen each moment with smooth transitions. Viola et al. [43] use viewpoint mutual information and a similar saliency-based EVMI to select the most expressive view for a specific focus of attention. When the user changes the focus of attention, the viewpoint is changed smoothly. Ruiz et al. [66] use a variation of projected saliency with the voxel information to compute the best viewpoint of a volume data set. Ruiz et al. [67] apply viewpoint Kullback–Leibler to compute automatic transfer functions. Itoh et al. [68] use a variant of viewpoint entropy for automatically selecting optimal viewpoints for visualizing spatio-temporal characteristics of trajectories on a crossroad using a space time cube. Tao et al. [69] apply viewpoint mutual information to 3D flow visualization to select best viewpoints, to decide how to cluster the streamlines and to create a camera path for automatic flow field exploration. Lee et al. [70] use normalized Shannon entropy to create a volumetric scalar entropy field to measure the complexity of a vector field. Maximum intensity projection of this volume is then used to obtain the maximum entropy viewpoints. Vázquez et al. [71] apply the use of minimum and maximum viewpoint entropy to the visualization of molecular structures to study their chemical and physical properties. Sarikaya et al. [72] use viewpoint selection techniques to identify features of interest on protein surfaces and to explore them efficiently.

González et al. [73] use viewpoint mutual information to compute the similarity between two 3D objects. Eitz et al. [74] use best view selection to retrieve a 3D object from a database given a 2D sketch. Li et al. [75] use viewpoint entropy to cluster the viewpoints and retrieve the most similar 3D object from a database given a 2D sketch. Bonaventura et al. [76] use viewpoint mutual information and information $I_{2}$ to compute the similarity between two 3D objects.

Castelló et al. [77] use viewpoint entropy, a variation of viewpoint Kullback–Leibler [78], viewpoint mutual information [79] and Tsallis generalization of viewpoint entropy and viewpoint mutual information [80], as measures for mesh simplification.

## 6. Conclusions

In this survey, we have reviewed a set of twenty-two measures for viewpoint selection, where eleven of these measures were not reviewed previously. We have extended a previous existing classification of viewpoint measures by Secord et al. [15], and we have implemented and compared them in a single framework, so as to allow for a fair comparison. As ground truth, we have used the Dutagaci et al. [14] user evaluation database. Our public framework allows for easily including any new measure for comparison, or use another database as ground-truth. The results short-listed five measures that effectively represent the viewpoint preferences of the users, between them three measures closely related to information theory. Finally, we have also presented the application fields that the different measures have been employed in, given that their utility could vary according to the purposes that they were designed for. In the future work, we will analyze the combination of some of the measures presented here. For instance, it is worth investigating a convex linear combination of the information-theoretic measures $I_{1}$, $I_{2}$, $I_{3}$, as it also provides a decomposition of mutual information. In addition, application of viewpoint measures to other fields such as augmented reality and 3D eye-tracking can be considered.

## Figures and Tables

**Table:** 

		
(**a**)	(**b**)	(**c**)
		
(**d**)	(**e**)	(**f**)

**Table:** 




**Table:** 

										
										
										
										
										
										
(a)	(b)	(c)	(d)	(e)	(f)	(g)	(h)	(i)	(j)	(k)
										
										
										
										
										
										
(l)	(m)	(n)	(o)	(p)	(q)	(r)	(s)	(t)	(u)	

**Table 1:** The most relevant notation symbols used in this paper.

*z*	polygon
Z	set of polygons
*v*	viewpoint
V	set of viewpoints
$a_{z}\left(v\right)$	projected area of polygon *z* from viewpoint *v*
$a_{t}\left(v\right)$	projected area of the model from viewpoint *v*
$vis_{z}\left(v\right)$	visibility of polygon *z* from viewpoint *v* (0 or 1)
$N_{p}$	number of polygons
*R*	number of pixels of the projected image
$A_{z}$	area of polygon *z*
$A_{t}$	total area of the model
p(z|v)	conditional probability of *z* given *v*
p(z)	probability of *z*
p(v|z)	conditional probability of *v* given *z*
p(v)	probability of *v*
H(V)	entropy of the set of viewpoints
H(Z)	entropy of the set of polygons
H(V|z)	conditional entropy of the set of viewpoints given polygon *z*
H(Z|v)	conditional entropy of the set of polygons given viewpoint *v*
slength(v)	silhouette length from viewpoint *v*
{h(α)}	normalized silhouette curvature histogram
α	turning angle bin
*a*	turning angle between two consecutive pixels
A	set of turning angles
$N_{a}$	number of turning angles
depth(v)	normalized maximum depth of the scene from viewpoint *v*
{h(d)}	normalized histogram of depths
*d*	depth bin
D	set of depth bins
$N_{v}$	number of neighbors of *v*
L(v)	size of the compression of the depth image corresponding to viewpoint *v*
$L(v_{i},v_{j})$	size of the compression of the concatenation of the depth images
	corresponding to viewpoints $v_{i}$ and $v_{j}$
$K_{i}$	curvature of vertex *i*
{h(b)}	normalized histogram of visible curvatures from viewpoint *v*
*b*	curvature bin
B	set of curvature bins
S(x)	saliency of vertex *x*

**Table 2:** List of measures (columns 1 and 2) grouped into five different categories with the corresponding names (columns 3, 4, and 5) used in surveys of Polonsky et al. [13], Dutagaci et al. [14], and Secord et al. [15], respectively. Column 6 indicates whether the best viewpoint corresponds to the highest (H) or the lowest (L) measure value. Column 7 shows whether the measure is sensitive (Y) to the polygonal discretization or not (N). Column 8 gives the main reference of the measure. Note: Character ‘-’ indicates that the measure was not tested in the corresponding survey.

#	Measure	Polonsky05	Dutagaci10	Secord11	V	D	Ref.
1	# Visible triangles	-	-	-	H	Y	[19]
2	Projected area	-	View area	*Idem*	H	N	[19]
3	Plemenos and Benayada	-	-	-	H	Y	[19]
4	Visibility ratio	*Idem*	Ratio of visible area	Surface visibility	H	N	[19]
5	Viewpoint entropy	Surface area entropy	Surface area entropy	*Idem*	H	Y	[21]
6	$I_{2}$	-	-	-	L	Y	[41]
7	VKL	-	-	-	L	N	[42]
8	VMI ($I_{1}$)	-	-	-	L	N	[35]
9	$I_{3}$	-	-	-	H	Y	[41]
10	Silhouette length	*Idem*	*Idem*	*Idem*	H	N	[13]
11	Silhouette entropy	*Idem*	*Idem*	-	H	N	[13]
12	Silhouette curvature	-	-	*Idem*	H	N	[46]
13	Silhouette curvature extrema	-	-	*Idem*	H	N	[15]
14	Stoev and Straßer	-	-	-	H	N	[23]
15	Maximum depth	-	-	Max depth	H	N	[23]
16	Depth distribution	-	-	*Idem*	H	N	[15]
17	Instability	-	-	-	L	Y	[35]
18	Depth-based visual stability	-	-	-	H	N	[49]
19	Curvature entropy	*Idem*	*Idem*	-	H	Y	[13]
20	Visible saliency	-	Mesh saliency	Mesh saliency	H	Y	[27]
21	Projected saliency	-	-	-	H	Y	[35]
22	Saliency-based EVMI	-	-	-	L	Y	[35]

**Table 3:** (Row 1) Reference of the paper. (Row 2) Measure used or inspired by. (Rows 3, 4, 5, 6, and 7) Field of application: scene exploration and camera placement (SE/CP), image-based modeling and rendering (IBMR), scientific visualization (SV), shape retrieval (SR), and mesh simplification (MS).

Reference	[62]	[36]	[57]	[63]	[58]	[37]	[59]	[48]	[64]	[65]	[71]	[43]	[77]	[73]	[78]	[79]	[35]	[60]	[66]	[80]	[74]	[61]	[75]	[69]	[72]	[76]	[70]	[68]
Measure	1	3	3,4	2	5	5	5	5,17	5	5,20	5	8,22	5	8	7	8	8	5	21	5,8	2,10,16	5	5	8	5	6,8	5	5
SE/CP		X	X		X		X										X	X				X						
IBMR	X			X		X																						
SV								X	X	X	X	X							X					X	X		X	X
SR														X							X		X			X		
MS													X		X	X				X								
